# Activity of Smurf2 Ubiquitin Ligase Is Regulated by the Wnt Pathway Protein Dishevelled

**DOI:** 10.3390/cells9051147

**Published:** 2020-05-07

**Authors:** Ondrej Bernatik, Petra Paclikova, Ranjani Sri Ganji, Vitezslav Bryja

**Affiliations:** 1 Institute of Experimental Biology, Faculty of Science, Masaryk University, 61137 Brno, Czech Republic; ondrej.bernatik@gmail.com (O.B.); 394465@mail.muni.cz (P.P.); ranjani.ganji@gmail.com (R.S.G.); 2 Department of Cytokinetics, Institute of Biophysics, Academy of Sciences of the Czech Republic, 61200 Brno, Czech Republic

**Keywords:** Smurf2, dishevelled, TGF-β/BMP signaling, Wnt signaling, prickle, ubiquitination

## Abstract

Wnt and BMP signaling pathways are two key molecular machineries regulating development and homeostasis. The efficient coordination of Wnt and BMP is essential in many developmental processes such as establishment of antero-posterior and dorso-ventral body axis, regulation of convergent extension, or development of various organ systems. SMAD ubiquitination regulatory factor (Smurf) family of E3 ubiquitin ligases are important and evolutionary conserved regulators of TGF-β/BMP signaling pathways. Smurf2 has been previously shown to regulate Wnt/planar cell polarity (PCP) signaling pathway by ubiquitinating Prickle1, one of the key components of PCP. We explored the role of Smurf2 in Wnt pathways in further detail and identified that Smurf2 is also a ubiquitin ligase of Dishevelled (DVL), the key cytoplasmic signal transducer in the Wnt pathway. Interestingly, the Smurf2 and DVL relationship expands beyond substrate-E3 ligase. We can show that DVL activates Smurf2, which allows Smurf2 to ubiquitinate its substrates from Wnt/PCP (Prickle1) as well as TGF-β/BMP (Smad2) pathways more efficiently. Using SMAD7 as an example of Smurf2 activator we show that DVL and SMAD7 both activates Smurf2 activity. In HEK293 cells the deficiency of DVL phenocopies absence of Smurf2 and leads to the increased phosphorylation of R-Smads. Smurf2-DVL connection provides a novel and intriguing point of crosstalk for Wnt and BMP pathways.

## 1. Introduction

Coordinated communication between cells is essential for the proper development and homeostasis. During development, multiple factors often act together in one place and in one cell. Analysis of molecular mechanism how individual signaling pathways crosstalk is thus important for understanding how regulatory circuits function. Here we focused on the regulation of TGF-β/BMP (transforming growth factor β/Bone morphogenic protein) and Wnt (Wingless-Int1) signaling pathways that without any doubt belong to the most important regulators of development. There are many developmental processes where tight coordination between Wnt and BMP pathway is required for the proper development. Examples of such processes include positional specification in the neural tube [1], specification of neural crest cells [2], or development of limbs [3].

SMAD (Suppressor of Mothers against Decapentaplegic) ubiquitination regulatory factor (Smurf) family of E3 ubiquitin ligases are important and evolutionary conserved regulators of TGF-β/BMP signaling pathways. The two members of Smurf protein family, Smurf1 and Smurf2, belong to a larger family of HECT type E3 ubiquitin ligases [4]. Smurf proteins regulate TGF-β/BMP pathways via ubiquitination of key signal transducers such as SMAD1, SMAD2, or SMAD5 [5,6], or TGF-β receptor I [7].

On top of their well described role in TGF-β/BMP pathways, Smurf ligases participate in several other processes. For example, Smurf2 was shown to be a part of regulatory circuit for spindle assembly checkpoint [8,9]. Smurf1 and Smurf2 influence cell migration by degradation of RhoA [10] or talin heads [11]. In addition, analysis of Smurf1/Smurf2 double knockout mice clearly demonstrated that Smurfs act as components of the Wnt/planar cell polarity (PCP) pathway [12]. Wnt/PCP pathway establishes cell polarity via asymmetric localization of two protein complexes consisting of Vangl and Prickle on one side, and Frizzled (Fzd), and Dishevelled (DVL) on the other side of a given cell [13]. It was reported that Smurf2 ubiquitinates Prickle1 in a PAR6- and Dishevelled (DVL)-dependent manner, which results in the locally restricted degradation of Prickle1 [12].

Despite the documented importance of Smurfs for the proper function of the PCP pathway, it is not clear how Smurf2 gets activated to selectively, in a DVL-dependent manner, target Prickle. In order to get further insight into this mechanism, we decided to take a closer look on the relationship of DVL and Smurf proteins. Surprisingly, we found out that DVL stimulates activity of Smurf2, not only towards Prickle, but also towards its known targets from TGF-β/BMP pathway. This study thus identifies DVL-Smurf2 interaction as a point for crosstalk of Wnt/PCP and TGF-β/BMP pathways. 

## 2. Materials and Methods

### 2.1. Cell Culture, Transfection, and Treatments

Wild type (wt) HEK-293t and all derived cell lines (HEK293t Smurf1/Smurf2 KO and HEK293t DVL1/DVL2/DVL3 KO) cells were propagated in DMEM/10% FCS/2 mM L-glutamine/50 units/mL penicillin/50 units/mL streptomycin. Cells (600,000 cells/well) were seeded into 6 well plate for ubiquitination analysis and into 24-well plate (200,000/well) for Western blotting analysis. The next day, cells were transfected using polyethylenimine (PEI) in a 3 μL PEI (2 mg/mL): 1 μg DNA ratio; medium was changed after 5 h of transfection. For Ubi assays we used 100–200 ng/plasmid per well, for rescue experiments 400 ng (370 ng of pcDNA + 30 ng of plasmid(s) encoding protein of interest per well (10 ng of each DVL expressing plasmid was used in triple DVL rescue experiments) if not specified otherwise. Cells were harvested for immunoblotting by lysis in 1× sample buffer (100 mM Tris/HCl (pH 6.8), 20% glycerol, 1% SDS). Protein concentration was measured by BIORAD DC protein assay according to manufacturer’s instructions. Before loading (20 μg/lane) β-mercaptoethanol (1% final concentration) and bromophenol blue (0.8 mg/mL final concentration) was added. Western blotting was done as published previously [14] Plasmids and antibodies used are listed in Table 1 and Table 2.

### 2.2. Ubiquitination Assay

Cells were pretreated for 8 h with epoxomicin (100 nM, Sigma-Aldrich E 3652) and then lysed by 600 µL of buffer containing 6 M guanidine hydrochloride, 0.1 M NaH_2_PO_4_/Na_2_HPO_4_ pH 8.0 (ratios calculated by http://clymer.altervista.org/buffers/phos2.html) 10 mM imidazole. Lysate was collected, sonicated, and heated to 95 °C for 6 min. Lysates were then centrifuged 16,200 g/20 min, and 500 μL of cleared lysate was used for incubation with magnetic His-beads (5 μL slurry) overnight (28-9799-17, GE-Healthcare). Pulldown samples were washed by buffer containing 8 M UREA, 0.1 M NaH_2_PO_4_/Na_2_HPO_4_ pH 6.3, 0.01 M Tris pH 8.0, 20 mM Imidazole, by using magnetic stand. After the last wash supernatant was completely removed and 100 μL of 1× sample buffer was added to the beads. Pulldown samples were heated to 95 °C for 6 min prior to loading. Lysates for input assessment were prepared by removal of SDS-PAGE incompatible guanidine-hydrochloride, by precipitation of the lysate (approximately 100 uL) by 900 μL of 100% ethanol overnight at −20 °C. Precipitated protein was then centrifuged at 16200 RCF for 20 min, supernatant was discarded, 500 μL of 90 % of ice-cold ethanol was added, vortexed, incubated at −20 °C for 20 min, centrifuged 16,200 RCF for 20 min, the supernatant was again discarded and the pellet was dried out and resuspended in 100 μL of 1× sample buffer.

### 2.3. Western Blot Quantification, Statistics

Western blot quantification was carried out using ImageJ distribution FIJI. Quantifications were normalized to average and test of statistical significance were performed using Students *t*-test by Graphpad prism (www.graphpad.com) statistics.

### 2.4. Generation of Smurf1 and Smurf2 KO HEK293t by CRISPR-Cas9

Guide RNAs were selected using http://crispr.mit.edu/ and cloned into pSpCas9(BB)-2A-GFP (PX458) [24]. Clones were selected using limited serial dilution and screened by WB for absence of Smurf1 and Smurf2 protein. gRNAs were used for Smurf1 and Smurf2 (Table 3).

## 3. Results

### 3.1. Smurf2 Is a DVL Ubiquitin Ligase That Targets DVL for Degradation

**Figure 1:**
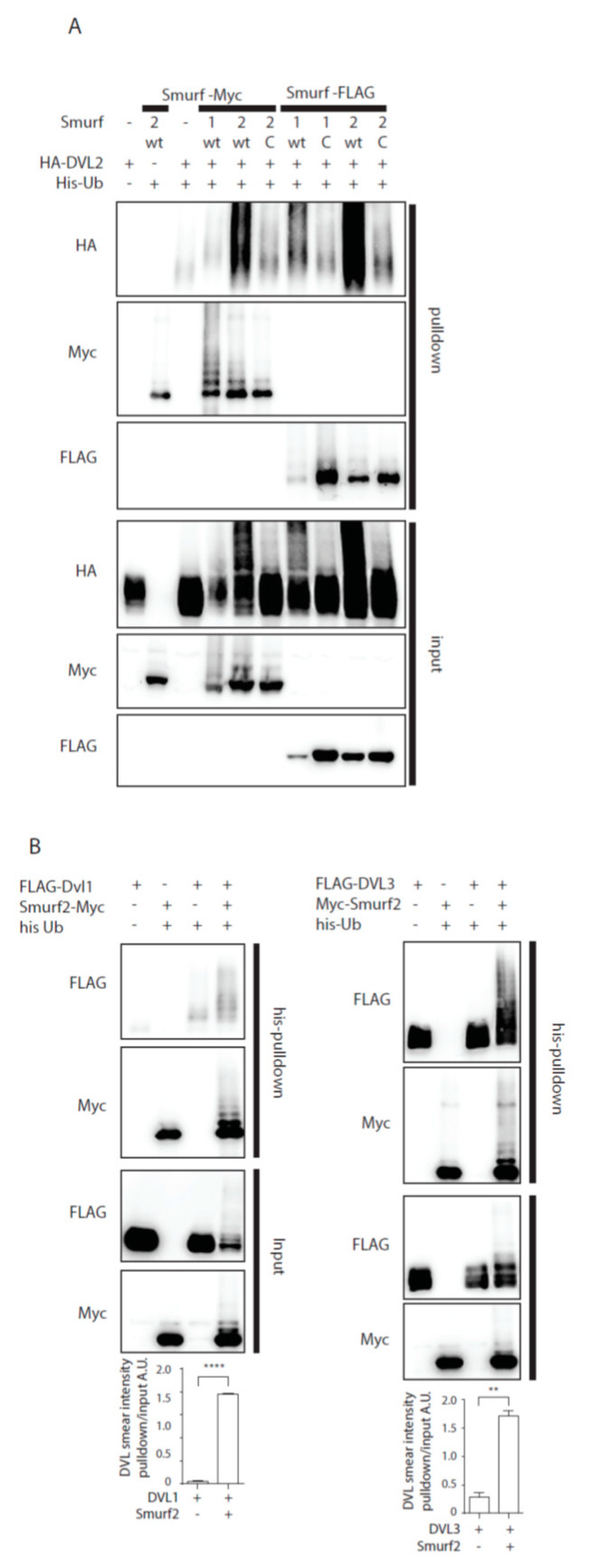

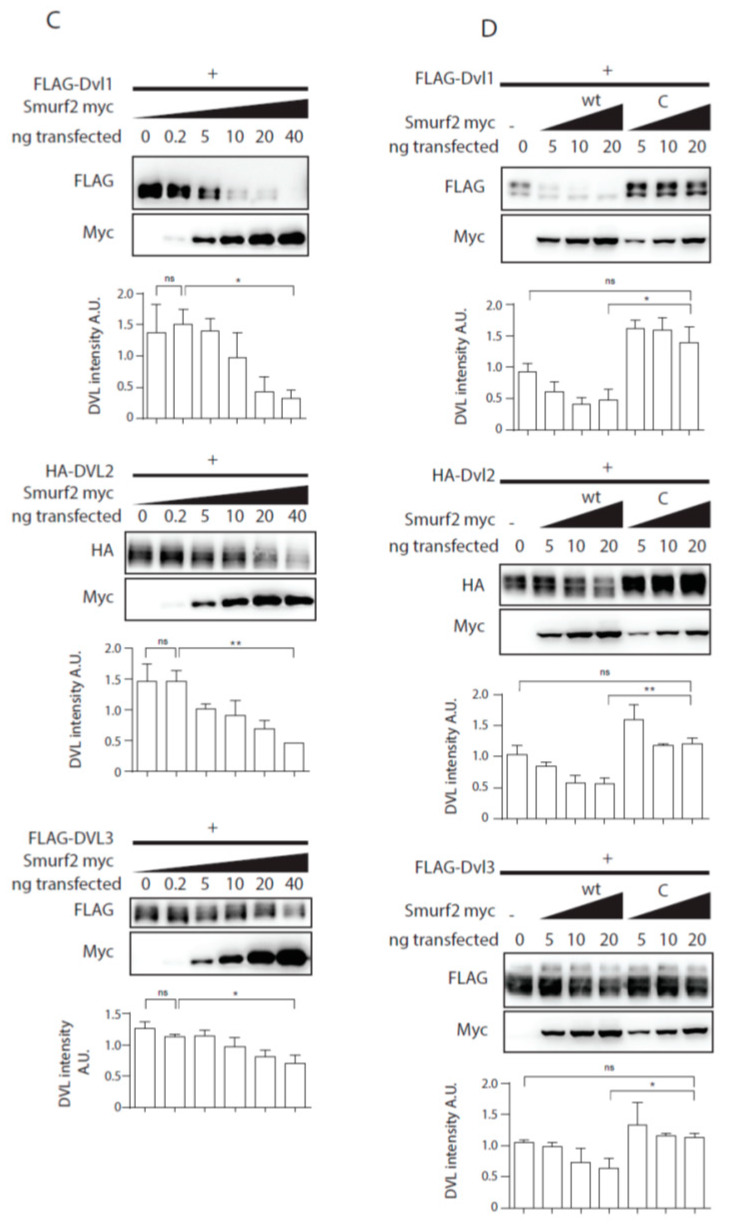
Smurf1 and Smurf2 ubiquitinate DVL. (**A**) DVL is a substrate for Smurf ubiquitin ligases. HEK293t cells were transfected with either wild type (wt) or catalytically inactive (C) versions of FLAG/myc-tagged Smurf1 or Smurf2, HA-DVL2 and His-Ubiquitin and subjected to ubiquitination assay. DVL2 is ubiquitinated by Smurf1 and Smurf2, ubiquitination of DVL2 is dependent on catalytic activity of Smurf1 or Smurf2 HECT domain. n = 3. (**B**) DVL1 and DVL3 are substrates for Smurf ubiquitin ligases. HEK293t cells were transfected with either wild type myc-tagged Smurf2, FLAG-DVL1, FLAD-DVL3 and His-Ubiquitin and subjected to ubiquitination assay. DVL1 and DVL3 are ubiquitinated by Smurf2. Quantification shows ratio of DVL smear above DVL band in pulldown to DVL band in the input. **** *p* < 0.001, ** *p* < 0.01. DVL1 n = 4, DVL3 n = 4. (**C**) Smurf2 causes degradation of DVL proteins. HEK293t cells were transfected with HA or FLAG tagged DVL isoforms and increasing doses (0, 0.2, 5, 10, 20, 40 ng) of wild type Smurf2. Smurf2 co-expression with DVL results in reduction of DVL levels in a dose-dependent manner. Graphs show quantification of intensity of DVL proteins in lines above, ** *p* < 0.01, * *p* < 0.05, ns = non-significant, n = 3, (**D**) HECT domain activity is indispensable for Smurf2 caused degradation of DVL proteins. HEK293t cells were transfected with HA or FLAG tagged DVL isoforms and increasing doses (5, 10, 20 ng) of wild type (wt) or catalytically inactive C716G (C) Smurf2. Wt Smurf2 reduced DVL protein levels in a dose-dependent manner in contrast to catalytically inactive C716G-Smurf2. Graphs show quantification of intensity of DVL proteins in lines above, ** *p* < 0.01, * *p* < 0.05, ns = non-significant, n = 3. Plasmids and antibodies used are listed in Table 1 and Table 2.

Previously published data identified Smurf2 as a component of the PCP machinery that mediates degradation of Prickle1 in a DVL-dependent manner [12]. However, it was not completely clear how DVL promotes Prickle 1 degradation and thus we decided to test the relationship of Smurf2 and DVL in further detail. As the first step in the analysis we tested whether Smurf ubiquitin ligases can ubiquitinate DVL. Overexpression of DVL2 with Smurf 1 or Smurf2 in HEK293t cells followed by the ubiquitination assay showed that Smurf2 and to a limited extent Smurf1 can catalyze modification of DVL2 by ubiquitin (Figure 1A). The ubiquitination of DVL2 by Smurf2 was dependent on the activity of Smurf2 HECT domain because the point mutant inactivating E3 ligase activity of Smurf2 (Smurf2-C716G, labeled “C”) was unable to promote ubiquitination (Figure 1A). This capacity of Smurf2 is not limited to DVL2, as we show in Figure 1B. Smurf2 also efficiently ubiquitinates other DVL isoforms such as DVL1 and DVL3. Smurf2-mediated ubiquitination of DVL1, DVL2, and DVL3 results in the downregulation of DVL protein levels. This trend is most pronounced in DVL2 and less detectable in DVL1 and DVL3 (Figure 1C). Again, the activity of Smurf2 HECT domain is essential, because inactive Smurf2-C716G is not able to reduce DVL1, DVL2, and DVL3 levels (Figure 1D).

### 3.2. Smurf2 Is Activated by DVL2

**Figure 2:**
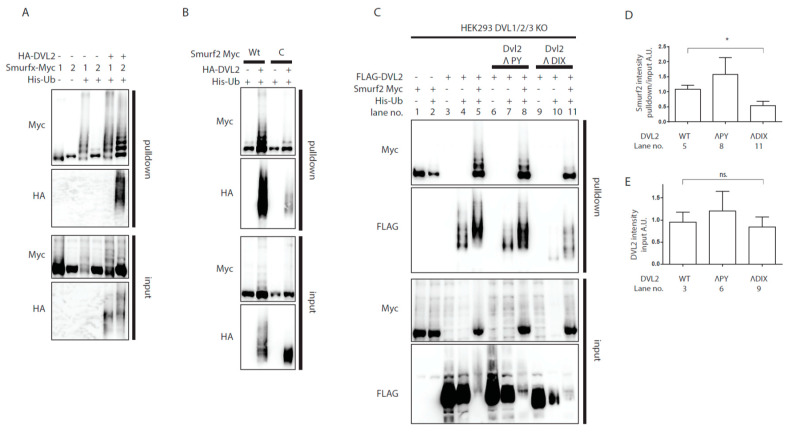
Analysis of Smurf2 autoubiquitination. (**A**,**B**) Wt HEK293t cells were transfected by indicated plasmids and subjected to ubiquitination assay. (**A**) DVL2 triggers Smurf2 autoubiquitination. Smurf proteins (Smurf1—1, Smurf2—2) were transfected. Smurf1 autoubiquitination is constitutive, whereas Smurf2 autoubiquitination is activated by DVL2 coexpression. n = 3. (**B**) Autoubiquitination of Smurf2 is dependent on activity of the HECT domain. Coexpression of DVL causes ubiquitination of wt Smurf2, minor ubiquitination of catalytically inactive Smurf2 (C) is detected, but not to the same extent as that of wt Smurf2. n = 3. (**C**) DVL1, DVL2, DVL3 triple KO HEK293 cells (HEK293 DVL1/2/3 KO) were transfected by indicated plasmids and subjected to ubiquitination assay. DVL2 PY motif is dispensable for activation of Smurf2 activity whereas deletion of entire DIX domain (aa1-90) of DVL2 hampers activation of Smurf2. n = 3. (**D**,**E**). Quantifications of Smurf2 and DVL2 in Figure 2C. (**D**) shows ratio of Smurf2 signal pulldown to input, lane numbers used for the quantification are indicated. (**E**) Shows DVL2 signal intensity in the input, lane numbers used for the quantification are indicated. * *p* < 0.05, ns. = non-significant. Plasmids and antibodies used are listed in Table 1 and Table 2.

Surprisingly, we noticed that co-expression of DVL clearly boosted ubiquitination of Smurf2 itself, visible as typical “ladder” in the ubiquitin pulldown probed for Smurf2 tag (FLAG or Myc) (Figure 1). Since it has been reported that Smurf2 is controlled by autoinhibition [25], we hypothesized that DVL is able to release the autoinhibition of Smurf2. In contrast to Smurf2, Smurf1 is not regulated by such autoinhibition [26] and can be used as a negative control. Indeed, the co-expression of DVL2 increased the ubiquitination of Smurf2 but not of Smurf1 (Figure 2A). In principle, increased ubiquitination of Smurf2 can be mediated by other E3 ligase brought into proximity by DVL2. To confirm that Smurf2 ubiquitination indeed represents autoubiquitination, we co-expressed DVL2 with wild-type (wt) or catalytically inactive (C) versions of Smurf2 (Figure 2B). Only Smurf2 with its intact HECT domain was more ubiquitinated in presence of exogenous DVL2, which suggests that DVL2 indeed interferes with Smurf2 autoinhibition (Figure 2B).

Interestingly, the capacity of DVL to prevent autoinhibition of E3 ligase activity was also shown for another member of the ubiquitin HECT E3 ligase family, WWP2 (WW domain-containing protein 2). DVL2 was capable to derepress autoinhibition of WWP2 HECT domain [27] and it was proposed that the activation of WWP2 by DVL depends (i) on the ability of DVL to polymerize via its DIX domain and (ii) on the interaction of PY motif of DVL with WW-domain of WWP2. We thus tested whether similar mechanism could apply to the action of DVL2 towards Smurf2. To rule out the influence of endogenous DVL, we used DVL1/DVL2/DVL3 ^-/-/-^ (DVL1/2/3 KO) HEK293t [28] cell line for these experiments. DVL2 lacking PY motif [27] was comparably potent activator of Smurf2 as WT DVL2 but on the other hand, DVL2 lacking the complete DIX domain failed to induce autoubiquitination of Smurf2 to the same extent as WT or PY motif lacking DVL2 (Figure 2C). These results suggest that similar DVL DIX domain-dependent activation of HECT domain might apply for Smurf2 and WWP2.

### 3.3. Smurf2 Activation by DVL2 Enhances Ubiquitination of Prickle1, but Not of Other PCP Proteins

**Figure 3:**
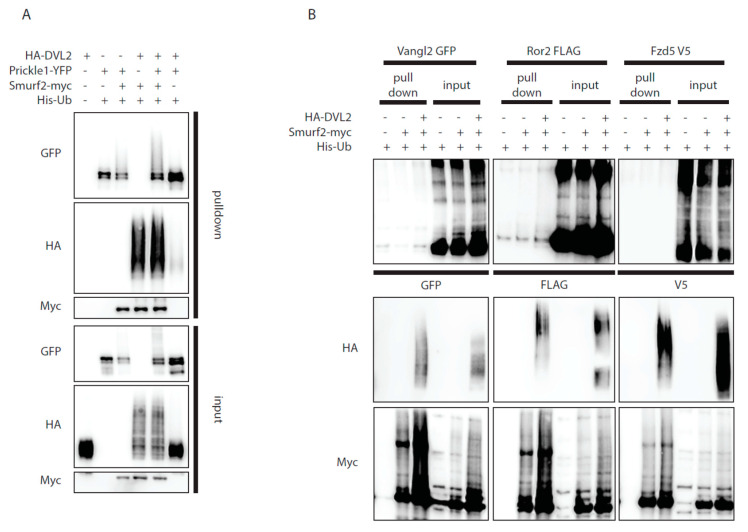
Dvl2 activates Smurf2 for ubiquitination of Prickle1, but not for other PCP proteins ubiquitination. (**A**,**B**) Wt HEK293t cells were transfected by indicated plasmids and subjected to ubiquitination assay. (**A**): Prickle1 is ubiquitinated by Smurf2 (third lane, GFP, pulldown), coexpression of DVL2 enhances Prickle1 ubiquitination (fifth lane, GFP, pulldown). n = 3. (**B**): None of the other PCP proteins tested (Vangl2, Ror2, Fzd5) are ubiquitinated by Smurf2. n = 2. Plasmids and antibodies used are listed in Table 1 and Table 2.

The ability of DVL2 to activate Smurf2 activity might provide a mechanistic explanation to how DVL controls local degradation of Prickle1, a known target of Smurf2 in the PCP pathway [12]. Indeed, in the presence of DVL2 Smurf2 got activated and triggered Prickle1 ubiquitination (Figure 3A). In order to test whether similar mechanism applies also for the other core Wnt/PCP proteins interacting with DVL we have co-expressed Smurf2 with Vangl2, Fzd5, and Ror2 in the presence and absence of exogenous DVL2. As shown in Figure 3B none of these proteins got ubiquitinated by Smurf2, regardless of DVL2 presence. This suggests that Prickle1 is the only core PCP pathway protein targeted by Smurf2 in a DVL-dependent manner, expanding the mechanism proposed by Narimatsu and colleagues [12].

### 3.4. Regulation of Smurf2 Activity by DVL2 Influences BMP Signaling

**Figure 4:**
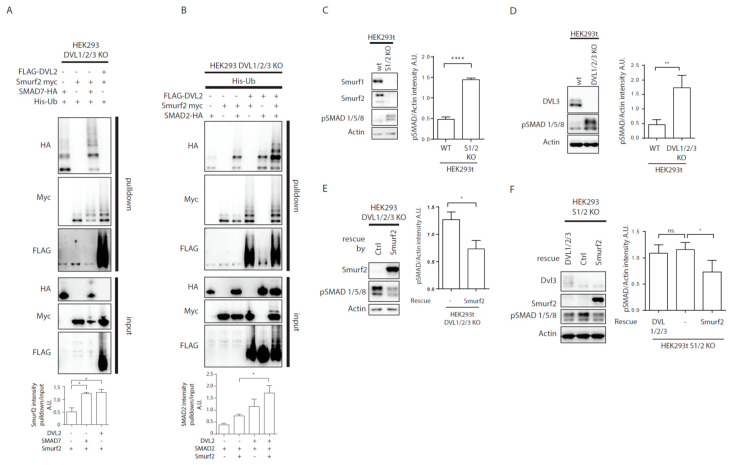
DVL regulates TGF-β/BMP pathways activity. (**A**,**B**) DVL KO HEK293t cells were transfected by the indicated plasmids and subjected to ubiquitination assay. (**A**) Both SMAD7 and DVL2 are activators of Smurf2. Graph shows quantification of ratio of pulldown to input intensity of Smurf2 protein in stated conditions, * *p* < 0.05, n = 3. (**B**) SMAD2 ubiquitination by Smurf2 is enhanced by DVL co-expression. Graph shows quantification of ratio of pulldown to input intensity of SMAD2 protein in stated conditions, * *p* < 0.05, n = 3. (**C**) Wt HEK293t and Smurf1 Smurf2 KO HEK29t3 (S1/2 KO) were seeded to equal density and collected after 48 h. Knock-out of Smurf1 and Smurf2 in HEK293 cells leads to overactivation of BMP pathway as measured by levels of pSMAD 1/5/8. Graph represents quantification of WB results, pSMAD1/5/8, and Actin intensities were normalized to average, n = 3, SEM, **** *p* < 0.0001. (**D**) Wt and DVL1/2/3 KO HEK293t cells were seeded to equal density and collected after 48 h. Knock-out of DVL1, DVL2, and DVL3 in HEK293 cells leads to overactivation of BMP pathway as measured by pSMAD 1/5/8 levels. Graph represents quantification of WB results, pSMAD1/5/8 and Actin intensities were normalized to average, n = 3, SEM, ** *p* < 0.01. (**E**) DVL1/2/3 KO HEK293t were transfected by pcDNA (-) as ctrl or Smurf2 expressing plasmid, and samples were collected after 48 h. Smurf2 inhibits BMP pathway activity in DVL1/2/3 KO HEK293. Graph represents quantification of WB results, pSMAD1/5/8, and Actin intensities were normalized to average, n = 3, SEM, * *p* < 0.05. (**F**) S1/2 KO HEK293t cells were seeded and were transfected by pcDNA (-) as ctrl, DVL1-FLAG, DVL2-FLAG, and DVL3-FLAG (DVL1/2/3) or Smurf2-Myc expressing plasmids. Smurf2 inhibits whereas DVL expression is unable to inhibit BMP pathway activity in S1/2 KO HEK293t cells. Graph represents quantification of WB results, pSMAD1/5/8, and Actin intensities were normalized to average, n = 3, SEM, * *p* < 0.05. Plasmids, antibodies and gRNAs used for CRISPR used are listed in Table 1, Table 2 and Table 3.

DVL is not the only protein reported to activate Smurf2. Similar activity has been well documented for SMAD7, a member of the inhibitory SMAD family; SMAD7 was shown to promote Smurf2 activity in order to target TGFβ receptor 1 for degradation and inhibit downstream signaling [7]. When we studied the potential of SMAD7 and DVL2 to induce Smurf2 autoubiquitination, we saw that SMAD7 and DVL are both efficient activators of Smurf2 (Figure 4A). This suggests that both SMAD7 and DVL2 can in principle inhibit TGF-β/BMP or Wnt-PCP pathways. Of note, SMAD7, known target of Smurf2, is ubiquitinated by Smurf2 in our assays, as was reported before [7], similarly to DVL, pointing on comparable mechanism of action of Smurf2 activation by these two proteins.

Originally, Smurf2 was identified as a negative regulator of TGF-β/BMP pathway [5,7,29]. The finding that DVL activated Smurf2 similarly to SMAD7 opened the possibility that DVL might act as an inhibitor of TGF-β/BMP pathway via activation of Smurf2. In order to test whether DVL2 indeed promotes Smurf2 activity towards substrates from TGF-β/BMP pathway we have decided to test SMAD2, a well reported target of Smurf2 in TGF-β/BMP pathway [6]. We overexpressed the combinations of DVL2, Smurf2, and SMAD2 in DVL1/2/3 KO DVL cells (to avoid interference with endogenous DVL proteins) and performed the ubiquitination assay. As shown in Figure 4B, the activity of Smurf2 towards SMAD2 was in the absence of DVL indistinguishable from the control but the level of SMAD2 ubiquitination triggered by Smurf2 increased when DVL2 was co-expressed. This suggests that DVL has the potential to regulate TGF-β/BMP signaling via regulation of Smurf2 activity.

To test whether DVL controls TGF-β/BMP signaling in an overexpression unperturbed system, we have decided to analyze DVL KO HEK293 cells [28] and compare them with Smurf1/2 double KO HEK293 cells (Smurf KO). Smurf KO cells were generated by CRISPR-Cas9 system in the same genetic background as DVL KO cells. As expected, Smurf KO HEK293 cells showed clearly higher SMAD1/5/8 phosphorylation (pS463/pS465) that is a hallmark of the BMP pathway activation (Figure 4C). We thus used the level of pSMAD1/5/8 as a readout and found significantly elevated levels also in DVL1/2/3 KO cells (Figure 4D). Overexpression of Smurf2 was able to reduce the activation of pSMAD1/5/8 in DVL1/2/3 KO cells (Figure 4E), suggesting that Smurf2 acts downstream of DVL via other Smurf2 activators such as SMAD7. However, the expression of DVL1/DVL2/DVL3 in Smurf KO HEK293 cells was unable to decrease the levels of pSMAD1/5/8, while it was rescued by reintroduction of Smurf2 (Figure 4F). This suggests that pSMAD1/5/8 regulation by DVL is fully dependent on Smurf2.

## 4. Discussion

Smurf ubiquitin ligases were identified in the late 90s as regulators of TGF-β/BMP signaling pathways. The two members of the family, Smurf1 and Smurf2, were shown to interact with and ubiquitinate R-SMADs (SMAD1, SMAD2, SMAD5) or TGF-β receptor I (Tβ-RI) [5,6,7] and their importance for TGF-β/BMP signaling was subsequently confirmed in vivo by study of *DSmurf* during *Drosophila* embryonic development [30]. In addition, Smurf proteins have been described to have additional functions. Several studies focused on the TGF-β/BMP independent regulatory functions of Smurfs found that Smurfs are also involved in control of cell cycle and cell migration [31]. Surprisingly, analysis of compound Smurf1^-/-^ Smurf2^-/-^ mouse mutants showed that these embryos exhibit typical Wnt/PCP phenotypes such as open neural tube and misoriented cochlear sensory hair cells that were observed in mouse mutants of many PCP genes like Vangl, Prickle, Fzd, or Celsr [1]. These genetic analyses placed Smurfs into the mammalian PCP pathway. The mode of action of Smurf proteins in the Wnt/PCP pathway was shown to be based on DVL- and PAR6-dependent and spatially restricted ubiquitination of the core PCP protein Prickle1. Prickle can thus be degraded when it appears in the proximity of DVL that localizes at the opposite site of a cell [12]. However, it was not clear if Prickle is the only target of Smurf and how DVL, on the molecular level, achieves activation of Smurf. Here we inspected how Smurf1 and Smurf2 influence DVL functions and found out that (i) Smurf2 and to a minor extent also Smurf1 can function as DVL ubiquitin ligases, (ii) DVL can activate Smurf2, and (iii) DVL can via Smurf regulate TGF-β/BMP signaling machinery due to novel interconnection of these two pathways on the level of DVL and Smurf2. 

Spatial separation of DVL/Frizzled and Vangl/Prickle complexes in the cell is an underlying principle for the establishment of planar cell polarity [13]. Activation of Smurf2 by DVL explains how DVL controls local degradation of Prickle1 in the cell regions “occupied” by DVL/Frizzled complex. Prickle1 seems to be the main target of Smurf2 in the PCP pathway because other PCP proteins that we tested (Ror2, Fzd5, Vangl2) were not ubiquitinated by Smurf2. This finding expands the mechanism proposed by Narimatsu and colleagues [12] and explains why DVL is required for Prickle ubiquitination by Smurf2.

DVL functions as a rail-switch between the Wnt/β-catenin (prototypical ligand: Wnt-3a) and the noncanonical Wnt (prototypical ligand: Wnt-5a) signaling pathways [2] are a function that is regulated by a set of posttranslational modifications and other protein components of the DVL complex [32,33,34,35,36]. DVL is also a target of several HECT type E3 ubiquitin ligases, namely ITCH (E3 ubiquitin-protein ligase Itchy homolog), that has been shown to degrade phophorylated DVL [37] or NEDD4L (neural precursor cell expressed developmentally down-regulated protein 4-like) that similarly to ITCH cause DVL degradation [38]. Different effect on DVL properties has been shown for another member of HECT domain E3 ligase family HUWE1, that inhibits the Wnt/β-catenin signaling by ubiquitination of the DVL DIX domain that does not lead to DVL degradation, but rather represses DVL multimerization and subsequent downstream signaling [39,40]. The last member of the HECT E3 family that has been to our knowledge added to the list of DVL ubiquitin ligases is WWP2 that was identified as DVL E3 ligase, which causes DVL degradation. The relationship between WWP2 and DVL extends beyond simple ligase-substrate connection—it was elegantly shown that DVL derepresses the autoinhibition of WWP2 HECT domain [27].

Similar to WWP2, Smurf2 HECT domain activity is autoinhibited by intramolecular interaction of its C2 to HECT domain [25]. Upon binding of adaptor proteins such as SMAD7, Smurf2 autoinhibition is released and Smurf2 can ubiquitinate its targets. To date only three proteins having Smurf2 activating properties were identified: SMAD7 that belongs to the family of I-SMADs, NDFIP2 (NEDD4 family interacting protein 2), and RNF11 (RING finger protein 11) [7,41,42]. Our study adds DVL2 to the list of proteins that can activate Smurf2. Considering the study by Mund and colleagues who described activation of WWP2 by DVL2, one can expect that DVL activates Smurf2 similarly to WWP2 [27]. Our data suggest that the mechanism is at least partly similar—in both cases, release of HECT autoinhibition requires DIX domain, a mediator of the DVL polymerization [18,43]. On the other hand, the PY sequence of DVL is dispensable for Smurf2 activation but necessary for WWP2 [27].

**Figure 5:**
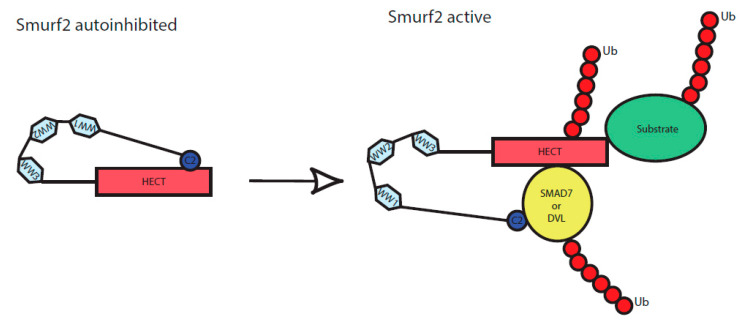
Scheme of Smurf2 activation by the binding of adaptor proteins. C2, WW1, WW2, WW3 and HECT represent individual protein domains of Smurf2. In an inactive state, Smurf2 is autoinhibited by intramolecular interaction of C2 to HECT domain, upon binding of the adaptor proteins (here represented by DVL or SMAD7) it reaches a novel conformation and gets activated. It can subsequently autoubiquitinate itself as well as its targets, including the activators (DVL, SMAD7) themselves.

Smurf2 is a well-characterized regulator of TGF-β/BMP signaling pathway. SMAD7 is known Smurf2 activating protein whose importance was extensively described for regulation of TGF-β/BMP activity [7,23,25]. We show that DVL activates Smurf2 similarly as SMAD7 and the action of DVL resembles RNF11, NDFIP2, and SMAD7, whose binding to Smurf2 causes the release of Smurf2 from the autoinhibited state and leads to subsequent ubiquitination of its targets [25,41]. We propose that similar mechanism applies to DVL that disinhibits Smurf2 HECT domain, which is otherwise blocked by intramolecular interaction, as schematized in Figure 5. Consequently, activation of Smurf2 via DVL resulted in the enhanced ubiquitination of TGF-β/BMP-relevant Smurf2 target SMAD2 [29,44]. Lack of DVL can thus lead to BMP pathway activation, that can be rescued by Smurf2 overexpression. The findings reported in this study provide evidence that DVL might act as a BMP pathway regulator that serves as a point of crosstalk for the Wnt and the TGF-β/BMP pathways.

## Figures and Tables

**Table 1:** Used plasmids [15,16,17,18,19,20,21,22,23].

FLAG-DVL3 wt	FLAG DVL1 wt	FLAG-DVL2 wt	FLAG-DVL2 ΔDIX
FLAG-DVL2 ΔPY	FLAG-Smurf1 wt	FLAG-Smurf1 C699A	FLAG-Smurf2 wt
FLAG-Smurf2 C716A	Smurf1–Myc	Smurf2-Myc wt	Smurf2-Myc CG
HA-DVL2 wt	Ror2-FLAG	Prickle	Vangl2
V5-Fzd5	SMAD7-HA (Addgene plasmid #11733)	SMAD2-HA (Addgene plasmid #14930)	

**Table 2:** Used antibodies.

Antibody	Catalogue Number	Manufacturer
FLAG M2	F1804	Merck
V5	R960-25	Invitrogen
c-Myc	C3956	Merck
c-Myc	Sc-40	Santa Cruz Biotechnology
DVL2	3216	Cell Signaling
DVL3	3218	Cell Signaling
DVL3	8027	Santa Cruz Biotechnology
HA11	MMS-101R	Covance
Smurf1	WH0057154M1	Merck
Smurf2	sc-25511	Santa Cruz Biotechnology
β-Actin	4970	Cell Signaling
pS463/pS465 SMAD 1/5/8	9511	Cell Signaling

**Table 3:** gRNA used for CRISPR.

	Guide 1	Guide 2
Smurf1, exon3	caccgTTGCAAAGATTGTCGTGGAT	aaacATCCACGACAATCTTTGCAAc
Smurf1, exon4	caccgATTCGATAACCATTAGCGTG	aaacCACGCTAATGGTTATCGAATc
Smurf2, exon6	caccgTCCTGTGCCTATTCGGTCTC	aaacGAGACCGAATAGGCACAGGAc
Smurf2, exon7	caccgTATAACAAGAACTACGCAAT	aaacATTGCGTAGTTCTTGTTATAc
